# Cross-linguistic influence of first language writing systems on brain responses to second language word reading in late bilinguals

**DOI:** 10.1002/brb3.153

**Published:** 2013-07-30

**Authors:** Satoru Yokoyama, Jungho Kim, Shinya Uchida, Tadao Miyamoto, Kei Yoshimoto, Ryuta Kawashima

**Affiliations:** 1 Department of Functional Brain Imaging Institute of Development, Aging, and Cancer, Tohoku University4-1 Seiryo-cho, Aoba-ku, Sendai, 980-8575, Japan; 2 Graduate School of Arts and Letters, Tohoku University27-1 Kawauchi, Aoba-ku, Sendai, 980-8576, Japan; 3 Department of Speech and Hearing Sciences, International University of Health and WelfareKitakanemaru 2600-1, Ohtawara city, Tochigi prefecture, 324-8501, Japan; 4 Graduate School of International Cultural Studies, Tohoku University41 Kawauchi, Aoba-ku, Sendai, 980-8576, Japan; 5 Smart Aging International Research Center Institute of Development, Aging, and Cancer, Tohoku University4-1 Seiryo-cho, Aoba-ku, Sendai, 980-8575, Japan

**Keywords:** Cross-linguistic, fMRI, orthography, second language, word reading, writing system

## Abstract

**Introduction** How human brains acquire second languages (L2) is one of the fundamental questions in neuroscience and language science. However, it is unclear whether the first language (L1) has a cross-linguistic influence on the processing of L2. **Methods** Here, we used functional magnetic resonance imaging to compare brain activities during L2 word reading tasks of phonographic Japanese Kana between two groups of learners of the Japanese language as their L2 and who had different orthographic backgrounds of their L1. For Chinese learners, a L1 of the Chinese language (Hanji) and a L2 of the Japanese Kana differed orthographically, whereas for Korean learners, a L1 of Korean Hangul and a L2 of Japanese Kana were similar. **Results** Our analysis revealed that, although proficiency and the age of acquisition did not differ between the two groups, Chinese learners showed greater activation of the left middle frontal gyrus than Korean learners during L2 word reading. **Conclusion** Our results provide evidence that strongly supported the hypothesis that cross-linguistic variations in orthography between L1 and L2 induce differential brain activation during L2 word reading, which has been proposed previously.

## Introduction

Although brain activities that are associated with first (L1) and second language (L2) comprehension have been examined and compared (Klein et al. 1995; Kim et al. 1997; Perani et al. 1998; Wartenburger et al. 2003; Rüschemeyer et al. 2005, 2006; Crinion et al. 2006; Yokoyama et al. 2006b, 2009; Abutalebi et al. 2008), less attention has been paid to the cross-linguistic influence of L1 on L2 processing (Jeong et al. 2007). As cross-linguistic variations among L1 induce distinct cortical activation patterns during L1 word recognition (Yokoyama et al. 2006a; Bick et al. 2010), differences between L1 and L2 may affect cortical activation during L2 processing. In particular, it has been shown that different neural substrates are associated with word reading between alphabetic (e.g., English), phonographic (e.g., Japanese), and logographic (e.g., Chinese) languages (Sakurai et al. 2000; Nakamura et al. 2005; Tan et al. 2005; Hu et al. 2010). The findings of these studies have led us to hypothesize that cross-linguistic variations in orthography between L1 and L2 induce differential brain activations during L2 word reading.

To the best of our knowledge, this possibility has been directly investigated in only a single functional magnetic resonance imaging (fMRI) study, in which the brain activities of native English speakers were scanned during a word reading task in English, while those of early Chinese-English bilinguals were scanned during word reading tasks in both Chinese and English (Tan et al. 2003). That study found that, despite processing two different language stimuli, the Chinese-English bilinguals exhibited similar activation patterns in the left middle frontal gyrus, which is well known as the brain region that is involved in the processing of Chinese Hanji words, during the processing of L1 (Chinese) and L2 (English) (French and Jacquet 2004; Siok et al. 2004, 2008). Additionally, despite processing identical English words, Chinese-English bilinguals and English natives displayed different cortical activation patterns. The authors interpreted these findings as an indication that the influence of L1 orthographic experience during development determines cortical activation during L2 word reading processing. However, because the brain activities of L2 learners of different L1 backgrounds were not directly compared, the study did not determine whether English natives process L1 and L2 words in an identical manner.

Here, we used fMRI to test the hypothesis of whether the influence of L1 orthographic experience during development determines cortical activation during L2 word reading processing. According to previous findings, Chinese native speakers use the left middle frontal gyrus to read Chinese Hanji words, which are logographic, as described above. If Tan et al. (2003)'s hypothesis is correct, the left middle frontal gyrus will be used, even when Chinese learners read words that are written in the phonographic system, which do not activate the left middle frontal gyrus in the case of L1 reading (Sakurai et al. 2000). In addition, if the hypothesis is correct, the left middle frontal gyrus will not be activated when native speakers who have a phonographic system as their L1 read L2 words that are written in the phonographic system. To this end, the current fMRI study compared the brain activities of Chinese and Korean learners during a word-reading task of Japanese Kana (L2). In terms of orthography, Korean Hangul is similar to Japanese Kana because they are both phonographic (i.e., a single letter is mapped onto a single sound unit but is generally not mapped onto a morpheme or meaning; the alphabetic system is included in this category), whereas Chinese Hanji is logographic (i.e., a single letter is mapped onto a single word or morpheme) and therefore markedly differs orthographically from Japanese Kana.

## Methods

### Participants

Ten native Chinese speakers (seven males and three females; mean age, 25.4 years) and seven native Korean speakers (three males and four females; mean age, 26.1 years) who learned Japanese as a L2 participated in this study. No significant differences in age were detected between the two groups of learners (ANOVA [analysis of variance]: *P* > 0.1). Because the age of acquisition (AOA) of words is critical in cortical representation (Wartenburger et al. 2003; Bloch et al. 2009), we controlled for the AOA between the Chinese (mean, 24.8) and Korean (mean, 22.7) learners (ANOVA: *P* > 0.1). The period of L2 learning did not differ between the Chinese (mean, 1.4 years; SD, 1.8) and Korean (mean, 3.4 years; SD, 4.3) learners (ANOVA: *P* > 0.1). All participants were either attending university or had graduated from university and were right-handed, as assessed with the Edinburgh Handedness Inventory (Oldfield 1971). None of the participants displayed any signs or had a previous history of medical or neurological diseases. Written informed consent was obtained from each subject in accordance with the guidelines approved by Tohoku University and the Helsinki Declaration of Human Rights, 1975. This study was approved by the ethical committee of Tohoku University Medical School.

The vocabulary proficiency levels of the two learner groups were assessed with part of the level-2 Japanese language proficiency test (only the vocabulary section), which was created by Japan Educational Exchanges and Services (Tokyo, Japan). No significant differences in test scores were detected between Chinese and Korean learners (mean scores [standard deviation {SD}]: Chinese learners, 58.1 [12.6]; Korean learners, 53.7 [21.3], ANOVA: *P* > 0.1).

### Experimental stimuli

As several studies have reported that different types of words show different brain activation patterns during reading (Yokoyama et al. 2006b), we included both nouns and verbs in this experimental study in order to exclude the possibility that the observed effects were specific to a certain word type. The stimuli were completely identical to those used in a previous study (Yokoyama et al. 2009). The Japanese writing system uses both phonographic Kana and logographic Kanji scripts. As the majority of Kanji characters are similar to those used in Chinese, we exclusively used Kana for the representation of Japanese stimuli in order to avoid the potential use of L1 Chinese knowledge by Chinese learners. The stimuli consisted of 60 actual words and 30 pseudowords. The pseudowords were constructed by exchanging a single consonant among actual words, and all were pronounceable. Thus, in order to perform the required task, the participants were required to recognize the target items, and there were no obvious visual cues to distinguish the actual words from the pseudowords. All of the stimuli were obtained from the level-3 and level-4 word lists of the Japanese language proficiency test, which are of a lower rank (i.e., fewer and considered less difficult) than those found on the level-2 test. Hence, our participants had already learned or previously encountered most of these words because they had all passed the level-2 test (see Participants).

### Experimental procedure

The experimental task that was required of the participants was designed to elicit a lexical decision with a blocked design. One block consisted of six trials, including pseudowords. Between blocks, subjects were instructed to look at a fixation cross that was displayed on the screen and to rest for 24 sec. Stimuli were presented on the screen for 2 sec, and the fixation cross was presented for 2 sec alternately in a block. Subjects were asked to silently read a visually presented word and to indicate whether the presented word was an actual word or a pseudoword by pressing a button with their index or middle finger, respectively, of their right hand. The accuracy rate and response time for all tasks that were used as behavioral data were collected with E-prime 1.0 software (Psychology Software Tools, Inc., Sharpsburg, PA) that was loaded on the Windows-based computer that was used in the presentation of the task stimuli.

### fMRI data acquisition

fMRI was performed at Tohoku University on a 1.5T Siemens Symphony scanner (Siemens Magnetom Symphony, Siemens AG, Erlangen, Germany). Head motion was minimized by placing pillows and cushions around the head. Thirty-two axial slices (4-mm thickness; field of view [FOV], 192 mm; in-plane resolution, 2 × 2 mm) were acquired every 3 sec during functional measurements (blood oxygen level-dependent sensitive gradient echo planar imaging sequence; repetition time [TR], 3000 msec; echo time [TE], 50 msec; flip angle, 90°) that were performed while the participants were doing the experimental reading task. Four initial scans were performed as dummy scans in order to equilibrate the state of magnetization, and these were excluded from the analysis. After the completion of functional imaging, anatomical images of T1-weighted images (1-mm thickness; FOV, 256 mm; data matrix, 192 × 224; TR, 1900 msec; TE, 3.93 msec) were also acquired from all participants.

### Data analysis

The obtained fMRI time-series data were analyzed with SPM8 software (Wellcome Institute of Cognitive Neurology, http://www.fil.ion.ucl.ac.uk/) that was implemented on MATLAB (The MathWorks, Inc., Natick, MA). Slice timing adjustment, realignment, spatial normalization to standard brain space, and smoothing with an isotropic Gaussian kernel of 8-mm full width at half maximum with the standard SPM method were conducted, and a high-pass frequency filter (128 sec) was applied. The time series was modeled and convolved with the hemodynamic response function. In order to investigate the influence of L1 orthography on L2 word reading, we directly compared brain activation for L2 word reading between Chinese and Korean learners by using two-sample *t*-tests and a small volume correction of the left middle frontal gyrus by using SPM. Therefore, the statistical threshold was *P* < 0.05 corrected. An anatomical mask image of the left middle frontal gyrus that was used in the small volume correction was made from WFU_pickatlas software (http://fmri.wfubmc.edu/software/PickAtlas). Additionally, in order to exclude the possibility that the results of the direct comparison were affected by the different proficiency levels between the two learner groups, we used vocabulary test scores as a confounding covariate in the two-sample *t*-test. Repeated measures (2 × 2) ANOVA was used to analyze the behavioral data (learner group × actual words/pseudowords).

## Results

Chinese (*n* = 10) and Korean (*n* = 7) learners were evaluated for their response times and accuracy rates in a task involving the reading of actual and pseudo Japanese (L2) words. The two groups of learners showed no significant differences in either their accuracy rates (*P* > 0.1) or response times (*P* > 0.1) in the L2 word-reading task. Both groups showed significantly longer response times to pseudowords compared to that to actual words (*P* < 0.05), although no differences in the accuracy rates were detected between word types (*P* > 0.1). The behavioral data of the two learner groups are summarized in Table 1.

**Table 1:** Behavioral data of the two learner groups for legal and pseudowords

Learner group	Legal	(SD)	Pseudo	(SD)
Accuracy rate (%)				
Korean	65	(17)	73	(12)
Chinese	73	(21)	83	(13)
Reaction time (msec)				
Korean	1667	(298)	1839	(377)
Chinese	1628	(395)	1898	(255)

SD, standard deviation.

**Figure 1:**
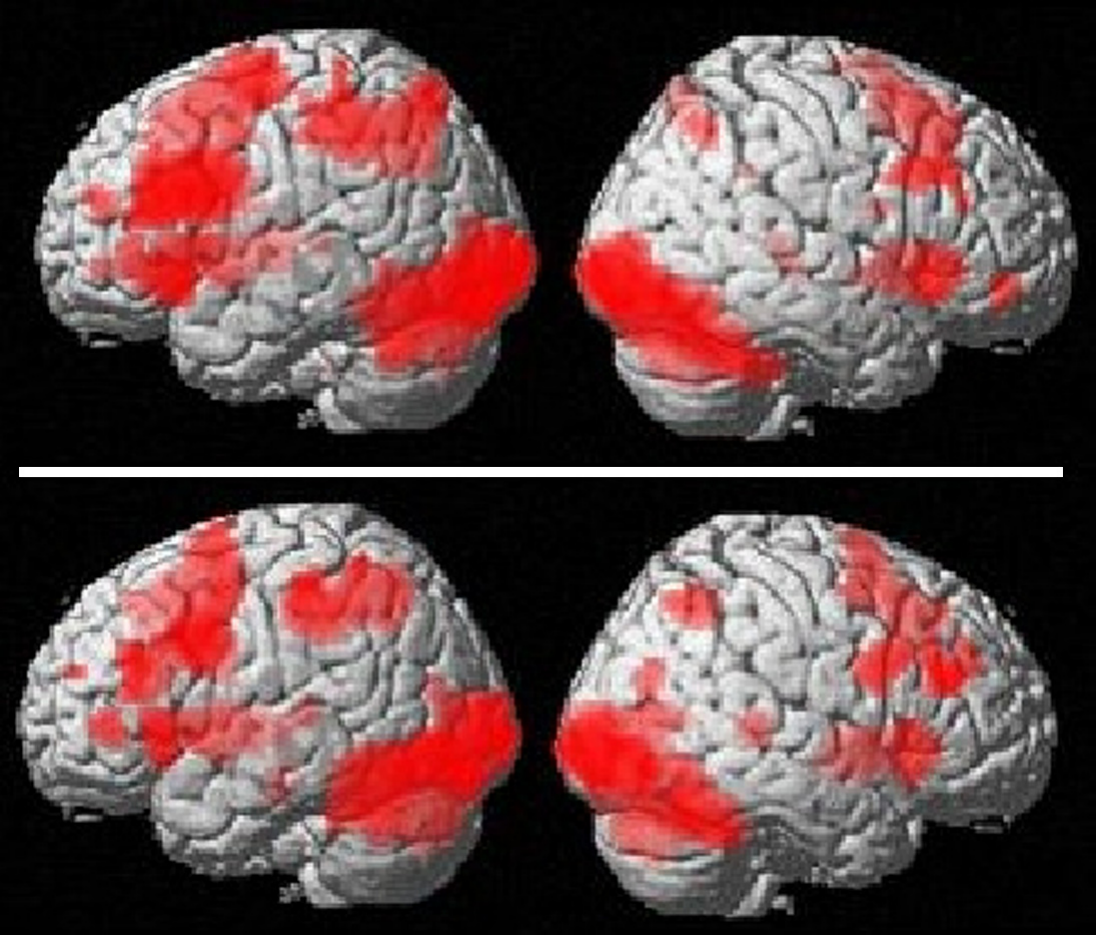
A surface rendering of word reading for Chinese and Korean learners. The upper and lower figures show the imaging results for Chinese and Korean learners, respectively. The significance threshold was set at *P* < 0.001 in height (uncorrected) for visual purposes.

**Figure 2:**
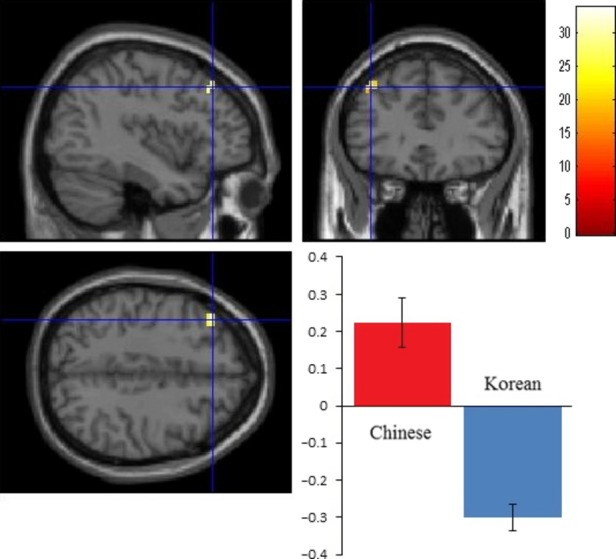
Differential brain activation during second language (L2) word reading between Chinese and Korean learners. Functional magnetic resonance imaging (fMRI) results of the left middle frontal gyrus. The graphs show the activation profiles for Chinese (red) and Korean (blue) learners. The statistical threshold was set at *P* < 0.05, corrected by small volume correction.

**Figure 3:**
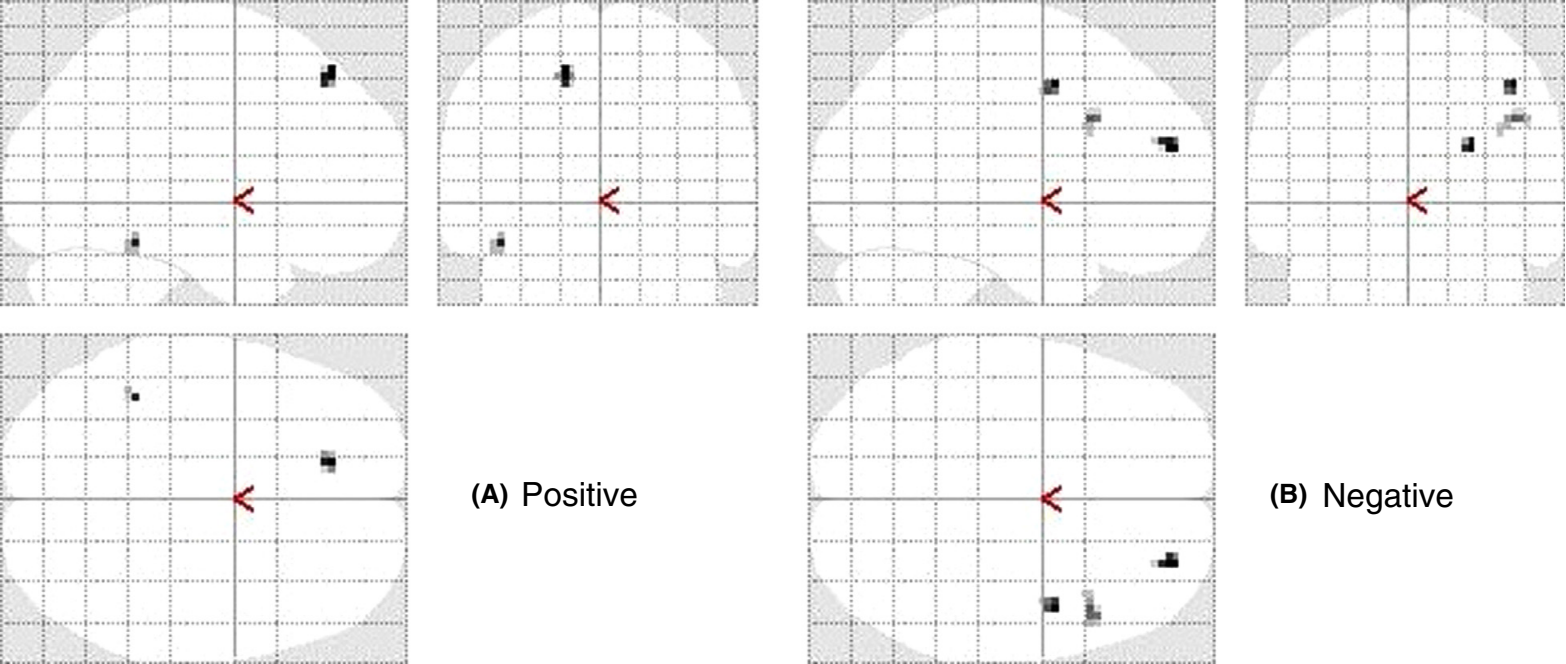
Brain activation during L2 word reading that was correlated with vocabulary test scores. The figures show the imaging results of brain activation that was correlated with vocabulary test scores, as evaluated by single regression and correlation analyses. (A) Positive correlation was detected in the left superior frontal gyrus and inferior temporal gyrus and (B) negative correlation was found in the right middle and inferior frontal gyri and precentral gyrus. The significance threshold was set at *P* < 0.001 in height (uncorrected) for visual purposes.

In the fMRI imaging results, the left parietal, bilateral frontal, temporal, and occipital cortices were significantly activated in both the Chinese and Korean learner groups (Fig. 1). In order to exclude the possibility that the results of the direct comparison were affected by the different proficiency levels in L2 word reading between the two learner groups, we used vocabulary test scores as a confounding covariate. In a direct comparison of the fMRI results between the two groups, Chinese learners showed significantly greater activation in the left middle frontal gyrus, and this activation survived at the *P* value (*P* < 0.05) that was corrected by the small volume correction (Fig. 2 and Table 2). In addition, to confirm that the left middle frontal activation we observed is not due to the L2 proficiency level in L2 word reading, we tested the correlation between the vocabulary test scores and brain activation. The vocabulary test scores positively correlated with brain activation of the left superior frontal gyrus and inferior temporal gyrus during the L2 word reading task and negatively correlated with the activation of the right middle and inferior frontal gyri and precentral gyrus (Fig. 3 and Table 2), indicating that the left middle frontal activation observed in the group comparison was not due to a proficiency effect of L2 word reading.

**Table 2:** Results of fMRI data analysis

Anatomical area	L/R	*F*	*k*	*P*	*x*	*y*	*z*
Chinese vs. Korean							
Middle frontal gyrus	L	33.81	11	0.001	−39	32	43

Anatomical area	L/R	*T*	*k*	*P*	*x*	*y*	*z*
Positive correlation							
Superior frontal gyrus	L	4.71	12	0.001	−15	41	55
Inferior temporal gyrus	L	4.47	6	0.001	−42	−43	−17
Negative correlation							
Middle frontal gyrus	R	4.5	10	0.001	27	53	22
Precentral gyrus	R	4.45	8	0.001	45	5	49
Opercular part of the inferior frontal gyrus	R	4.13	13	0.001	45	20	34

L, left; R, right; *k*, cluster size.

## Discussion

In the present fMRI study, we tested the hypothesis that L1 orthographic experience during development determines cortical activation during L2 word reading processing. Notably, we found that the Chinese learners showed significantly greater activation in the left middle frontal gyrus than Korean learners during L2 Japanese phonographic word reading (Fig. 2 and Table 2). Our findings strongly supported Tan et al. (2003)'s hypothesis that cross-linguistic differences in L1 orthography affect the cortical processing of L2 word reading in L2 learners.

Because we controlled for differences in age, AOA of Japanese (L2), and L2 vocabulary proficiency level, which are known to affect brain activation during L2 processing (see Methods), the observed activation patterns of the left middle frontal region were independent of the activation that was elicited by these factors. Additionally, no differences in the behavioral performances in the reading task were identified between the two groups. Because these factors cannot account for the differences in brain activation, our results indicated that differential cortical activation was induced by orthographic differences in the L1 writing system, namely phonographic Hangul for Korean and logographic Hanji for Chinese. Although the number of subjects that was included in our study was limited due to the highly specialized population, previous brain activation studies that have a similar purpose and design have used a similar number of subjects (Wartenburger et al. 2003; Yokoyama et al. 2009). However, we detected statistically robust differences with correction for multiple comparisons between the two learner groups with a random-effect model, which enabled us to generalize the observed results. Hence, our results can be interpreted as an indication that cross-linguistic differences in L1 orthography affect the cortical processing of L2 word reading in L2 learners.

Further, it is important to discuss the role of the left middle frontal gyrus during L2 word reading in learners who have experience using a logographic writing system such as L1. In fact, there are two main hypotheses for the mechanism. The first is that the difference observed here was caused by a processing demand during L2 word reading when differences existed between the orthography of L1 and L2. Actually, we found different activities in the left middle frontal gyrus between Chinese and Korean learners (Fig. 1), and this region is related to processing demand or control for L2 processing (Pillai et al. 2004). However, it has been previously demonstrated that, compared to Chinese subjects with dyslexia, normal Chinese subjects show better behavioral performance and greater activation of the left middle frontal gyrus during Chinese word reading (Hu et al. 2010). This finding indicates that the left middle frontal gyrus activation that was observed in this study during word reading was not due to neural effort because normal Chinese subjects require less effort and exhibit more activation in this region than do Chinese subjects with dyslexia during reading. Here, no differences in task performance or vocabulary proficiency test scores were detected between the Chinese and Korean learners. In addition, the brain regions that were activated and correlated with vocabulary proficiency test scores differed from those activated in the direct comparison between the two groups of learners (Figs. 2, 3 and Table 2), suggesting that different processing demands activated regions other than the left middle frontal gyrus. Thus, this possible interpretation was negated.

The second hypothesis is that the experience of L1 orthography tunes cortical activation during L2 word reading processing (Tan et al. 2003). In several previous studies, the left middle frontal gyrus was specifically active for the reading of logographic characters (Tan et al. 2003, 2005; Siok et al. 2004, 2008; Hu et al. 2010). In particular, Tan et al. (2005) proposed that the left middle frontal gyrus acts as a phonological processer for logographic characters, whereas the left temporoparietal regions are activated for alphabetic characters using meta-analysis methods. Theoretically, a single logographic character has both semantic and phonological information, whereas a single phonographic character, including the alphabet, has essentially no semantic information. Hence, in logographic writing systems, orthography-to-phonology mapping processes are necessary, which are based on long-term memory. The left middle frontal gyrus may play a role in such a process (Tan et al. 2005). In contrast, in phonographic writing systems, because several characters are combined in a single word, the grapheme-to-phoneme conversion process is necessary to read the word, which is based on rule-based computation. Additionally, Tan et al. (2003) proposed that cross-linguistic differences in L1 orthography affect the cortical processing of L2 word reading in L2 learners; that is, L1 orthographic experience tunes cortical mechanisms for L2 word reading. Our results support this hypothesis because L2 word reading written by phonographic characters (i.e., Kana in the current study) activates the left middle frontal gyrus in Chinese learners who have experience with logographic writing systems such as L1. Additionally, L2 phonographic reading does not activate the left middle frontal gyrus in Korean learners who have experience with phonographic writing systems (i.e., Hungul) such as L1.

Before concluding, our results interestingly showed that vocabulary test scores negatively correlated with the activation of several frontal regions during the L2 word reading task (Figs. 2, 3 and Table 2). Previous studies have reported that proficient L2 learners show less activation in the frontal region than less proficient L2 learners during L2 processing (Chee et al. 2001; Wartenburger et al. 2003; Yokoyama et al. 2009). In addition, a recent longitudinal neuroimaging study of L2 processing has reported that, when L2 proficiency level increases, frontal activation decreases during L2 word processing (Stein et al. 2009). Hence, our results of the negative correlation between vocabulary test scores and frontal activation may reflect less activation of the frontal regions with more efficient frontal control of L2 word reading. Another interpretation is that less activation of the frontal regions may be the result of having more L2 vocabulary because more vocabulary enables the efficient use of cortical resources, which causes a reduction in the activation of the frontal regions (Prat and Just 2011). Of course, this is speculative, and it is hard to determine which interpretation is appropriate to explain our results. Thus, further studies are necessary.

In conclusion, the present fMRI study investigated whether L1 orthography influenced L2 word reading by Chinese and Korean L2 learners of the L2 of Japanese. Although the behavioral performances and AOA did not markedly differ between the two groups, Chinese learners showed greater activation in the left middle frontal gyrus than Korean learners did. These activation results were independent of the activation that was elicited by differences in proficiency levels between the two groups, suggesting that this activity of the left middle frontal gyrus was not due to the different processing demands between the two groups. Our results strongly support Tan et al. (2003)'s hypothesis that the experience of L1 orthography determines cortical activation during L2 word reading processing.

## References

[b1] Abutalebi J, Annoni JM, Zimine I, Pegna AJ, Seghier ML, Lee-Jahnke H (2008). Language control and lexical competition in bilinguals: an event-related FMRI study. Cereb. Cortex.

[b2] Bick AS, Goelman G, Frost R (2010). Hebrew brain vs. English brain: language modulates the way it is processed. J. Cogn. Neurosci.

[b3] Bloch C, Kaiser A, Kuenzli E, Zappatore D, Haller S, Franceschini R (2009). The age of second language acquisition determines the variability in activation elicited by narration in three languages in Broca's and Wernicke's area. Neuropsychologia.

[b4] Chee MW, Hon N, Lee HL, Soon CS (2001). Relative language proficiency modulates BOLD signal change when bilinguals perform semantic judgments. Blood oxygen level dependent. Neuroimage.

[b5] Crinion J, Turner R, Grogan A, Hanakawa T, Noppeney U, Devlin JT (2006). Language control in the bilingual brain. Science.

[b6] French RM, Jacquet M (2004). Understanding bilingual memory: models and data. Trends Cogn. Sci.

[b7] Hu W, Lee HL, Zhang Q, Liu T, Geng LB, Seghier ML (2010). Developmental dyslexia in Chinese and English populations: dissociating the effect of dyslexia from language differences. Brain.

[b8] Jeong H, Sugiura M, Sassa Y, Yokoyama S, Horie K, Sato S (2007). Cross-linguistic influence on brain activation during second language processing: an fMRI study. Biling. (Camb. Engl.).

[b9] Kim KH, Relkin NR, Lee KM, Hirsch J (1997). Distinct cortical areas associated with native and second languages. Nature.

[b10] Klein D, Milner B, Zatorre RJ, Meyer E, Evans AC (1995). The neural substrates underlying word generation: a bilingual functional-imaging study. Proc. Natl. Acad. Sci. USA.

[b11] Nakamura K, Dehaene S, Jobert A, Kouider D, Le Bihan S (2005). Subliminal convergence of Kanji and Kana words: further evidence for functional parcellation of the posterior temporal cortex in visual word perception. J. Cogn. Neurosci.

[b12] Oldfield RC (1971). The assessment and analysis of handedness: the Edinburgh inventory. Neuropsychologia.

[b13] Perani D, Paulesu E, Galles NS, Dupoux E, Dehaene S, Bettinardi V (1998). The bilingual brain. Proficiency and age of acquisition of the second language. Brain.

[b14] Pillai JJ, Allison JD, Sethuraman S, Araque JM, Thiruvaiyaru D, Ison CB (2004). Functional MR imaging study of language-related differences in bilingual cerebellar activation. Am. J. Neuroradiol.

[b15] Prat CS, Just MA (2011). Exploring the neural dynamics underpinning individual differences in sentence comprehension. Cereb. Cortex.

[b16] Rüschemeyer SA, Fiebach CJ, Kempe V, Friederici AD (2005). Processing lexical semantic and syntactic information in first and second language: fMRI evidence from German and Russian. Hum. Brain Mapp.

[b17] Rüschemeyer SA, Zysset S, Friederici AD (2006). Native and non-native reading of sentences: an fMRI experiment. Neuroimage.

[b18] Sakurai Y, Momose T, Iwata M, Sudo Y, Ohtomo K, Kanazawa I (2000). Different cortical activity in reading of Kanji words, Kana words and Kana nonwords. Cogn. Brain Res.

[b19] Siok WT, Perfetti CA, Jin Z, Tan LH (2004). Biological abnormality of impaired reading is constrained by culture. Nature.

[b20] Siok WT, Niu Z, Jin Z, Perfetti CA, Tan LH (2008). A structural-functional basis for dyslexia in the cortex of Chinese readers. Proc. Natl. Acad. Sci. USA.

[b21] Stein M, Federspiel A, Koenig T, Wirth M, Lehmann C, Wiest R (2009). Reduced frontal activation with increasing 2nd language proficiency. Neuropsychologia.

[b22] Tan LH, Spinks JA, Feng CM, Siok WT, Perfetti CA, Xiong J (2003). Neural systems of second language reading are shaped by native language. Hum. Brain Mapp.

[b23] Tan LH, Laird AR, Li K, Fox PT (2005). Neuroanatomical correlates of phonological processing of Chinese characters and alphabetic words: a meta-analysis. Hum. Brain Mapp.

[b24] Wartenburger I, Heekeren HR, Abutalebi J, Cappa SF, Villringer A, Perani D (2003). Early setting of grammatical processing in the bilingual brain. Neuron.

[b25] Yokoyama S, Miyamoto T, Riera J, Kim J, Akitsuki Y, Iwata K (2006a). Cortical mechanisms involved in the processing of verbs: an fMRI study. J. Cogn. Neurosci.

[b26] Yokoyama S, Okamoto H, Miyamoto T, Yoshimoto K, Kim J, Iwata K (2006b). Cortical activation in the processing of passive sentences in L1 and L2: an fMRI study. Neuroimage.

[b27] Yokoyama S, Kim J, Uchida SY, Miyamoto T, Yoshimoto K, Riera J (2009). Left middle temporal deactivation caused by insufficient second language word comprehension by Chinese-Japanese bilinguals. J. Neurolinguistics.

